# Factors influencing the intention to engage in cervical cancer preventive behavior in human papillomavirus-infected women: a cross-sectional survey

**DOI:** 10.4069/kjwhn.2023.11.13.2

**Published:** 2023-12-28

**Authors:** Bogyeong Song, So Young Choi

**Affiliations:** 1 Women's Medicine Center, Changwon Hanmaeum Hospital, Changwon, Korea; 2 College of Nursing Institute of Health Science, Gyeongsang National University, Jinju, Korea

**Keywords:** Human papillomavirus, Prevention, Self efficacy, Uncertainty, Uterine cervical neoplasms

## Abstract

**Purpose:**

This study investigated the influence of cervical cancer knowledge, human papillomavirus (HPV) knowledge, self-efficacy, and uncertainty on the intention to engage in cervical cancer preventive behavior in HPV-infected women.

**Methods:**

This descriptive correlational study was conducted among 129 adult women aged 20 to 65 years who received positive HPV results at a general hospital in Changwon, Korea. The dataset was analyzed using descriptive statistics, the independent t-test, analysis of variance, the Pearson correlation coefficient, and multiple regression.

**Results:**

The mean score for the intention to engage in cervical cancer preventive behavior was high (4.43±0.65). This intention was significantly different according to age at first sexual intercourse (F=7.38, *p*=.001), HPV type (F=4.79, *p*=.010), vaccination (t=3.19, *p*=.002), and condom use (t=3.03, *p*=.003). The intention to engage in cervical cancer preventive behavior showed significant, weak-to-moderate positive correlations with HPV knowledge (r=.22, *p*=.012) and self-efficacy (r=.42, *p*<.001). Self-efficacy (β=.46, *p*<.001), first sexual intercourse at >20 years (β=.45, *p*<.001), first sexual intercourse at 20-24 years (β=.29, *p*=.018), HPV high- and low-risk group infection (β=.26, *p*=.019), HPV high-risk group infection (β=.26, *p*=.026) and vaccination (β=.21, *p*=.007) significantly influenced the intention to engage in cervical cancer preventive behavior. These variables explained 34.6% of variance in this intention.

**Conclusion:**

It is necessary to develop a program that effectively conveys accurate information about cervical cancer prevention to HPV-infected women and helps them enhance self-efficacy to boost the intention to engage in cervical cancer preventive behavior.

## Introduction

The human papillomavirus (HPV), which is primarily transmitted through sexual contact, has been recognized as a significant cause of cervical cancer [1]. To date, more than 200 types of HPV have been identified [2], with high-risk strains such as HPV-16 and HPV-18 being strongly linked to genital cancers, especially cervical cancer [3]. According to data from the Korea Central Cancer Registry in 2022 [4], cervical cancer constituted 1.2% of all cancer cases in Korea in 2020, ranking as the 10th most common cancer among women. The 2021 Cancer Trends Report from the National Cancer Center [5] indicates that each year, over 3,000 Korean women are diagnosed with cervical cancer, leading to approximately 900 deaths. Over 95% of these cancers have been connected to HPV [6], and nearly 100% of invasive cervical cancer cases are associated with HPV infection [7].

Preventive behavior refers to actions taken by individuals who perceive themselves to be healthy in order to detect or prevent disease before symptoms appear [8]. It is of the utmost importance to increase the intention to engage in cervical cancer preventive behavior among HPV-infected women.

HPV vaccination and regular screening are essential preventive measures against cervical cancer [9]. The vaccine is effective in preventing new HPV infections, even after initial exposure, but it cannot cure existing infections or related diseases [6]. It is also important to avoid smoking, which is a known risk factor, and to be cautious with long-term use of oral contraceptives, as they can increase the risk of cervical cancer by 1.2 to 1.5 times [9]. Additionally, limiting the number of sexual partners and consistently using condoms during intercourse are recommended [1]. Research has indicated that various factors, including perceived benefits and the severity of health beliefs related to vaccination, contraceptive use, and HPV testing experience, enhance cervical cancer prevention behaviors among nurses [10]. A study focusing on nursing students found that those with a higher intention to engage in cervical cancer preventive behavior were more likely to participate in cervical cancer screening [11]. Furthermore, research involving Korean women in their 20s revealed that more conservative sexual attitudes were associated with increased preventive behavior [12]. In a study on female university students, higher levels of self-efficacy and fear were associated with a stronger intention to engage in preventive behavior [13].

Providing information about cancer to foster positive attitude changes has been shown to be an effective strategy for promoting the practice of cervical cancer prevention behaviors [14]. Research indicates that greater knowledge about cervical cancer is associated with more consistent engagement in preventive behavior [15]. However, despite the importance of intending to engage in such behaviors, adult Korean women generally possess limited knowledge about the disease. For instance, only 41.7% are aware of the connection between HPV infection and cervical cancer [16], and even nurses’ understanding of HPV is notably deficient [10].

Women who test positive for HPV often experience significant anxiety and uncertainty due to the associated risk of developing cervical cancer [17]. This anxiety extends to concerns about the potential effects on pregnancy and childbirth [18]. Studies involving gastric cancer and hemodialysis patients have demonstrated that uncertainty can influence the intention to engage in preventive behavior, with greater uncertainty correlating with a reduction in health-promoting actions [19]. Additionally, hemodialysis patients tend to show lower adherence to prescribed sick role behaviors when they face greater uncertainty and have a more serious perception of their illness [20]. However, research has yet to explore the impact of uncertainty on the intentions of HPV-infected women to engage in preventive behavior.

Self-efficacy is a critical factor in increasing the intention to engage in preventive behavior [21]. It influences not only health-promoting actions but also acts as an essential bridge between knowledge and behavior [22]. Elevated self-efficacy is associated with improved health behaviors, and interventions designed to boost self-efficacy can lead to positive behavioral modifications, ultimately enhancing health outcomes [23]. Moreover, heightened self-efficacy correlates with more regular participation in cervical cancer prevention behaviors [24] and has been recognized as a mediator in developing the intention to prevent cervical cancer [13].

Therefore, this study aimed to assess the levels of cervical cancer and HPV knowledge, self-efficacy, and uncertainty in HPV-infected women, as well as their effects on the intention to engage in cervical cancer preventive behavior. The goal was to provide foundational data that could inform the development of programs to improve the intention of HPV-infected women to engage in cervical cancer preventive behavior.

The purpose of this study was to identify factors influencing HPV-infected women’s intention to engage in cervical cancer preventive behavior. These behaviors are defined as voluntary and diligent actions aimed at cancer prevention. The study had the following specific aims: first, to investigate how participants’ characteristics influence their intention to engage in cervical cancer preventive behavior; second, to assess levels of intention, cervical cancer knowledge, HPV knowledge, self-efficacy, and uncertainty among participants; third, to identify correlations between these variables and preventive behavior intention; and fourth, to determine the factors that influenced participants’ intentions to engage in such behaviors.

## Methods

**Ethical statement:** This study was approved by the Institutional Review Board of Changwon Hanmaeum Hospital (No. H2023-001-1) in Changwon, Korea. Informed consent was obtained from the participants.

### Research design

The purpose of this descriptive correlational study was to identify factors influencing the intention to engage in cervical cancer preventive behavior among women infected with HPV. This study adhered to the STROBE reporting guidelines (https://www.strobe-statement.org/).

### Participants

This study enrolled adult women between the ages of 20 and 65 years who tested positive for HPV at the obstetrics and gynecology department of a general hospital in Changwon, Korea. Participants were selected through convenience sampling and included those who could communicate in Korean, understood and consented to the study’s objectives, and were reachable within one week of receiving their HPV test results. Women diagnosed with carcinoma in situ or cervical cancer were excluded from the study. The required sample size was calculated using G*Power 3.1.9.2, based on a previous study [25]. The parameters set for the calculation included a significance level of .05, a medium effect size of .15, a power of 80%, and nine predictors: cervical cancer knowledge, HPV knowledge, uncertainty, self-efficacy, smoking, age at first sexual intercourse, number of sexual partners, condom use, and HPV vaccination. The calculation indicated that a sample size of 114 would be sufficient for multiple regression analysis. To account for a potential 20% dropout rate, the study aimed to enroll 143 participants. Ultimately, the final analysis was conducted on 129 cases after excluding 14 responses deemed inadequate.

### Instruments

All tools in this study were used after receiving approval via e-mail from the corresponding developer and translator. A structured questionnaire with 99 items was used, covering topics such as the intention to engage in cervical cancer preventive behavior, cervical cancer knowledge, HPV knowledge, uncertainty, self-efficacy, and both general and HPV-related characteristics.

#### Intention to engage in cervical cancer preventive behavior

The intention to engage in cervical cancer preventive behavior was assessed using a tool developed by Ko [26], drawing on the study of Yoo et al. [27] on the intention to prevent novel flu and Han’s research [28] on early cancer screening promotion messages targeting Korean and Japanese women aged 30 to 59 years. This instrument consists of six items, each addressing a distinct aspect of cervical cancer prevention: seeking information about prevention, consulting with a physician, undergoing regular screenings, recommending screenings to others, getting vaccinated, and advocating for HPV vaccination for others. Responses are measured on a 5-point Likert scale, ranging from 1 (“strongly disagree”) to 5 (“strongly agree”), where higher scores reflect a stronger intention to engage in preventive behavior. For this study, the mean scores were calculated, with possible values ranging from 1 to 5. The tool demonstrated high reliability, with Cronbach’s α of .88 in the initial study [10] and .81 in the current study.

#### Cervical cancer knowledge

Cervical cancer knowledge was assessed using a tool modified and expanded by Kim and Park [29], based on the study of Lee and Lee [30]. This instrument comprises eight items: four addressing the risk factors for cervical cancer, one regarding its incidence, one concerning symptoms, one related to diagnosis, and one about prognosis. Responses were categorized as “true,” “false,” or “do not know.” Correct responses were awarded 1 point, while incorrect or “do not know” answers received no points. A higher aggregate score, ranging from 0 to 8, reflected a more comprehensive understanding of cervical cancer. At the time of its development, the tool demonstrated a Cronbach’s α of .83 [29], and in this study, the Kuder-Richardson Formula 20 (KR-20) reliability coefficient was .61.

#### Human papillomavirus knowledge

HPV knowledge was assessed using a 20-item tool developed by Kim and An [31]. This tool encompasses a range of topics, including the association between HPV and cervical cancer, symptoms of HPV, the distinction between low-risk and high-risk types, correlations with latency periods, prognosis, and immunity, the ages at which HPV is most prevalent, modes of transmission, methods of examination and diagnosis, strategies for prevention and treatment, and the risk of congenital infections. The scoring approach mirrored that of the cervical cancer knowledge assessment, with scores ranging from 0 to 20, where higher scores indicated a more comprehensive understanding of HPV. The reliability of the instrument at the time of its development [31] was reflected by a Cronbach’s α of .87, and in the current study, the KR-20 reliability coefficient was .84.

#### Uncertainty

The Mishel Uncertainty in Illness Scale Community Form (MUIS-C) [32], as translated by Chung et al. [33], was utilized to measure uncertainty. This instrument comprises 23 items categorized into four subdomains: ambiguity, complexity, inconsistency, and unpredictability. Respondents rate each item using a 5-point Likert scale, where 1 signifies “not at all” and 5 indicates “very much.” Higher scores denote increased uncertainty. In the analysis, average scores were calculated, with possible values ranging from 1 to 5. The MUIS-C’s original reliability was reported with Cronbach’s α values ranging from .91 to .93 [32]; Cronbach’s α was .85 in the study by Chung et al. [33], and it was .82 in the current study.

#### Self-efficacy

Self-efficacy was measured using a Korean version of a 24-item health management self-efficacy scale, which was translated and subjected to factor analysis by Lee et al. [34]. This adapted version originated from the 28-item scale developed by Becker et al. [35]. The scale encompasses six subdomains: exercise management (eight items), disease management (four items), emotional management (three items), nutrition management (three items), stress management (three items), and health behavior management (three items). Responses are scored on a 5-point Likert scale, ranging from 1 (“strongly disagree”) to 5 (“strongly agree”), with higher scores denoting increased self-efficacy. For this study, the mean scores were calculated, which could vary from 1 to 5. The reliability of the instrument was confirmed with a Cronbach’s α of .91 in the study by Lee et al. [34] and .92 in the current study.

#### General and human papillomavirus-related characteristics

The general characteristics of the participants included seven items: age, marital status, educational level, job, average monthly household income, drinking, and smoking. HPV-related characteristics included 11 items: menopause status, age at first sexual intercourse, number of sexual partners during lifetime, current condom use, frequency of sexual intercourse, frequency of Pap tests, HPV vaccination status, induced abortion, childbirth, cervical cancer test results, and HPV types.

### Data collection

Women who visited the obstetrics and gynecology outpatient clinic and tested positive for HPV were recruited for the study between February and April 2023. The researcher explained the purpose of the study and the survey details over the phone to potential participants. After participants agreed to take part in the study, an online survey link was sent to them. The online survey began with a description of the study, and clicking “agree” was considered to indicate consent. Participants who completed the survey received a mobile beverage coupon worth 4 US dollars as a token of appreciation.

### Data analysis

IBM SPSS for Windows ver. 27.0 (IBM Corp., Armonk, NY, USA) was used to analyze the data.

The general and HPV-related characteristics of participants were analyzed using frequency, percentage, and mean with standard deviation. We examined differences in knowledge of cervical cancer and HPV, levels of uncertainty, self-efficacy, and the intention to engage in cervical cancer preventive behavior according to these characteristics. This examination was conducted using the t-test and analysis of variance, with subsequent post-hoc analysis performed using the Scheffé test. To analyze the levels of knowledge about cervical cancer and HPV, uncertainty, self-efficacy, and the intention to engage in cervical cancer preventive behavior, we employed the mean and standard deviation. Pearson’s correlation coefficients were utilized to explore the relationships between knowledge of cervical cancer and HPV, uncertainty, self-efficacy, and the intention to engage in cervical cancer preventive behavior. Finally, multiple regression analysis was applied to identify factors that influenced the intention to engage in cervical cancer preventive behavior.

## Results

### Differences in cervical cancer preventive behavior intentions based on general and human papillomavirus-related characteristics

The average age of the participants was 37.96±9.31 years. Among them, 84 individuals (65.1%) were married, with the most common level of education being a bachelor’s degree or higher, as reported by 58 participants (45.0%). A significant majority, 97 participants (75.2%), were employed. The average monthly household income was 4.84±2.94 million Korean won, which is approximately 3,657.05±2,224.10 US dollars. Alcohol consumption was reported by 78 participants (60.5%), while 115 (89.1%) indicated that they were nonsmokers (Table 1).

In terms of HPV-related characteristics, 118 of the participants (91.5%) were premenopausal. The average age of first sexual intercourse was 20.80±3.09 years, with 39 participants (30.2%) reporting their first sexual encounter before the age of 20 years. The average number of lifetime sexual partners was 4.74±4.20, with the most common response being one partner, as reported by 24 participants (18.6%). A majority, 95 (73.6%), did not use condoms, and the most frequently reported frequency of sexual intercourse was less than once a month, at 48.1%. Regarding preventive health measures, 45 (34.9%) of the participants underwent annual Pap tests, and 66 (51.2%) had received HPV vaccinations. A total of 90 participants (69.8%) reported no history of induced abortion, and high-risk HPV types were detected in 66 participants (51.2%) (Table 1).

The intention to engage in cervical cancer preventive behavior showed significant relationships with the following factors: age at first sexual intercourse (F=7.38, *p*=.001), HPV type (F=4.79, *p*=.010), vaccination status (t=3.19, *p*=.002), and condom use (t=3.03, *p*=.003). According to the Scheffé post-hoc test, participants who first engaged in sexual intercourse before the age of 20 years or between the ages of 20 and 24 years had a higher intention for such behavior than those who were 25 years or older at the time of first sexual intercourse. Participants with high-risk HPV types or a combination of high- and low-risk types exhibited higher intentions than those with only low-risk types. Furthermore, those who had been vaccinated or used condoms showed a greater intention to engage in cervical cancer preventive behavior (Table 1).

### Levels of intention to engage in cervical cancer preventive behavior, cervical cancer knowledge, human papillomavirus knowledge, uncertainty, and self-efficacy

Participants exhibited a high intention to engage in cervical cancer preventive behavior, with an average score of 4.43±0.65 on a 1 to 5 scale. Knowledge of cervical cancer was somewhat above average, scoring 4.87±1.86 on a 0 to 8 scale. HPV knowledge was moderate, with an average score of 10.04±4.36 on a 0 to 20 scale. The level of uncertainty was slightly below average at 2.42±0.52 on a 1 to 5 scale, while self-efficacy exceeded the average, registering at 3.90±0.70 on a 1 to 5 scale (Table 2).

### Correlations between intention to engage in cervical cancer preventive behavior, cervical cancer and human papillomavirus knowledge, uncertainty, and self-efficacy

The intention to engage in cervical cancer preventive behavior was positively associated with both HPV knowledge (r=.22, *p*=.012) and self-efficacy (r=.42, *p*<.001). Knowledge about cervical cancer was positively correlated with HPV knowledge (r=.63, *p*<.001) and inversely correlated with uncertainty (r=–.21, *p*=.016). Furthermore, HPV knowledge was negatively correlated with uncertainty (r=–.21, *p*=.015), and uncertainty was inversely related to self-efficacy (r=–.33, *p*<.001) (Table 3).

### Factors influencing the intention to engage in cervical cancer preventive behavior

Multiple regression analysis was used to identify factors that influence the intention to engage in cervical cancer prevention behaviors. The independent variables considered were age at first sexual intercourse, HPV type, vaccination status, condom use, and knowledge of cervical cancer and HPV, as well as levels of uncertainty and self-efficacy. Categorical variables such as age at first sexual intercourse (with ≥25 years as the reference), HPV type (with low-risk as the reference), vaccination status (with unvaccinated as the reference), and condom use (with non-use as the reference) were dummy-coded for analysis. The Durbin-Watson statistic was 1.643, suggesting the absence of autocorrelation in the residuals. Furthermore, the standardized residuals fell within the range of 3, indicating a normal distribution of errors. Multicollinearity was not a concern, as indicated by tolerance values between .346 and .873 and variance inflation factors ranging from 1.146 to 2.893. The analysis identified several significant factors that influence the intention to engage in cervical cancer preventive behavior for cervical cancer. These factors, in order of significance, included self-efficacy (β=.46, *p*<.001), first engaging in sexual intercourse before the age of 20 years (β=.45, *p*<.001) or between the ages of 20 and 24 years (β=.29, *p*=.018), infection with both high- and low-risk HPV types (β=.26, *p*=.019), infection with high-risk HPV types alone (β=.26, *p*=.026), and vaccination (β=.21, *p*=.007). Consequently, higher self-efficacy, earlier age at first sexual intercourse, and infection with high-risk HPV types or both high- and low-risk types, as opposed to only low-risk types, along with being vaccinated, significantly increased the intention to engage in cervical cancer preventive behavior. Collectively, these factors accounted for 34.6% of the variance in the intention to engage in cervical cancer preventive behavior (Table 4).

## Discussion

This study aimed to assess the levels of cervical cancer and HPV-related knowledge, self-efficacy, and uncertainty among women infected with HPV, as well as the factors influencing their intention to engage in cervical cancer preventive behavior, with the goal of developing programs to increase such intentions in HPV-infected women. The results indicated that self-efficacy, age at first sexual intercourse, HPV infection type, and vaccination status were all significant determinants of their preventive behavior intention.

In this study, participants demonstrated higher cervical cancer knowledge scores than those reported in similar studies using the same assessment tool. For instance, a study involving nursing students [11] reported scores of 4.83 for those who had undergone a Pap test and 3.98 for those who had not. In contrast, female university students scored 3.74 in another study [36]. Likewise, the HPV knowledge scores of participants in this study exceeded those found in other research: 2.74 for married immigrant women [37], 5.33 for female university students [36], and 7.98 for nurses [10]. The elevated level of knowledge among HPV-infected women in this study may be attributed to their heightened interest in HPV and cervical cancer, which likely motivates them to actively seek information from various sources, including medical professionals. However, given the potential risk of cervical cancer in HPV-infected women, their knowledge level, while comparatively high, is still not sufficient. Therefore, systematic education focused on HPV management and cervical cancer prevention for HPV-infected women is necessary.

This study found that participants had an average uncertainty score of 2.42 out of a possible 5 points, reflecting a moderate degree of uncertainty. This finding aligns with results from previous research using the same measurement tool, which reported scores of 2.47 in gastrectomy patients with gastric cancer [19], 2.67 in patients undergoing hemodialysis [20], and 2.52 in female thyroid cancer patients [38]. However, there is a scarcity of research on uncertainty in women infected with HPV. Given the unpredictable nature of treatment outcomes and the potential progression to cervical cancer, it is crucial to alleviate uncertainty in these women. Providing education about HPV and cervical cancer can play a significant role in this effort by offering accurate knowledge and information.

The average score for the intention to engage in cervical cancer preventive behavior among the study participants was 4.43. Although there are no directly comparable prior studies focusing on HPV-infected women, similar research utilizing the same measurement tool has yielded varying results. In Ko’s study [26], women between the ages of 20 and 50 years had an average score of 3.62. A separate study involving nurses reported an average score of 3.55 [10], and female university students scored an average of 4.25 in research conducted by Nguyen and Lee [13]. Compared to these outcomes, the intention of HPV-infected women in the current study to participate in preventive behavior seems to be relatively high.

The factors influencing the intention to engage in cervical cancer preventive behavior were self-efficacy, age at first sexual intercourse, HPV type, and vaccination status, listed in order of impact. Previous research [39] has shown that self-efficacy exerts a stronger influence on health-promoting behaviors, preventive behavior, and sick role behaviors than other variables. It also affects university students’ intentions to adopt preventive behavior for emerging infectious diseases [40]. In a study with female university students, Nguyen and Lee [13] identified self-efficacy as a mediating factor for the intention to practice cervical cancer prevention. Similarly, Ma et al. [24] found self-efficacy to be a highly influential factor among Chinese individuals in their twenties. This study corroborates these findings, highlighting self-efficacy as the most significant factor, in line with numerous previous studies. These results indicate that confidence in one’s ability to take appropriate action in specific situations can significantly affect the willingness to engage in health-promoting behaviors. Consequently, it is essential to develop strategies that enhance self-efficacy to increase women’s intention to engage in cervical cancer preventive behavior, particularly among those infected with HPV.

Regarding the HPV-related characteristics of the participants, initiating sexual intercourse between the ages of 20 and 24 years, or before the age of 20 years, being infected with high-risk HPV or both high- and low-risk types, and receiving the HPV vaccine were all identified as factors influencing the intention to engage in cervical cancer preventive behavior. However, the limited amount of prior research on HPV-infected women makes direct comparisons with these findings challenging. Since 2016, Korea has implemented the Healthy Women’s First Step Clinic Program since 2016, providing free HPV vaccinations to 12-year-old female adolescents [41]. In general, receiving the complete vaccine series before becoming sexually active is the most effective method for preventing cervical cancer. Nevertheless, vaccination remains beneficial for those who are sexually active, already infected with HPV, or older, as it can prevent new HPV infections and reinfection with existing HPV strains, thereby reducing the risk of cancer [6]. Therefore, it is imperative to provide HPV-infected women with accurate information about cervical cancer prevention, educate them on specific preventive actions, and emphasize the importance and benefits of HPV vaccination.

HPV infection is the most direct and significant cause of cervical cancer among its various causes. Women who test positive for HPV often face psychological and social challenges, including shock, confusion, anxiety, and fear of the disease [41]. However, cervical cancer can be prevented through regular screenings, HPV vaccination, and adherence to sexual health preventive behavior. Early detection and treatment are key to reducing mortality rates [9]. Therefore, it is vital to promote the intention to engage in cervical cancer preventive behavior among women with HPV. This study highlights the importance of boosting self-efficacy and developing effective educational programs for prevention. Furthermore, because HPV is a sexually transmitted infection, it is particularly important to address infected women’s negative emotions and the societal stigma associated with HPV. Such efforts will contribute to the creation of a supportive environment, encouraging these women to actively engage in cervical cancer preventive behavior.

Interestingly, this study found that knowledge of cervical cancer and HPV did not significantly influence participants’ intention to engage in cervical cancer preventive behavior. This finding aligns with previous research [12,26] indicating that knowledge alone is not sufficient to motivate preventive behavior. Additionally, these findings suggest that, while knowledge is a necessary component for increasing cervical cancer preventive behavior, knowledge alone is insufficient to induce the intention to engage in such behavior. While the KR-20 reliability score for the cervical cancer knowledge tool in this study was low (.61), it was still identified as influential. Consequently, there is a need for further development of a more reliable cervical cancer knowledge tool for future research.

This study included a wide range of participants, ranging from women newly diagnosed with HPV or those with a relatively brief infection duration, to women with a history of HPV infection who were undergoing regular follow-up over a prolonged period. However, a limitation of this study is the lack of consideration for the duration of the participants’ HPV infection. Research on diabetes patients [42] has shown that the length of time since diagnosis is associated with statistically significant differences in self-care behaviors. Specifically, patients who had been diagnosed with diabetes for over 10 years exhibited better self-care practices than those diagnosed for less than five years. Consequently, future studies should take the time since diagnosis into account.

Another limitation of the study is the difficulty of generalizing the findings to all women affected by HPV, as the sample consisted solely of HPV-infected women from a single general hospital in a specific region. Therefore, further research is needed to investigate the timing and duration of HPV infection across a broader demographic. Additionally, follow-up studies are essential to create and validate effective cervical cancer prevention programs tailored to women with HPV infection.

Most HPV research in Korea has centered on specific groups such as nurses, university students, and unmarried women. However, there has been a notable lack of investigation into the lived experiences of women who have contracted HPV. This study holds significance as it sheds light on the motivations behind cervical cancer preventive behavior in women with HPV. These insights provide crucial foundational data that will inform the creation of effective cervical cancer prevention programs.

## Figures and Tables

**Table 1.** Differences in the intention of cervical cancer preventive behavior according to participants’ characteristics (N=129)

Characteristics	Categories	Mean±SD or n (%)	Intention to engage in cervical cancer preventive behavior		
			Mean±SD	t or F	*p* ^†^
Age (year)		37.96±9.31			
	20–29	24 (18.6)	27.58±3.32	1.92	.129
	30–39	51 (39.5)	27.06±3.66		
	40–49	37 (28.7)	25.40±4.59		
	≥50	17 (13.2)	26.70±3.49		
Marital status	No	45 (34.9)	27.38±3.55	1.58	.116
	Yes	84 (65.1)	26.24±4.07		
Educational level	High school	27 (20.9)	25.26±4.36	2.16	.120
	College	44 (34.1)	27.07±3.65		
	≥University	58 (45.0)	26.95±3.82		
Job	Yes	97 (75.2)	26.66±3.90	0.12	.904
	No	32 (24.8)	26.56±4.04		
Monthly income (KRW)		484.01±294.36			
	3 million	38 (29.5)	27.18±3.56	1.89	.134
	3 million–3.9 million	29 (22.5)	26.93±3.86		
	4 million–4.9 million	47 (36.4)	25.62±4.38		
	>5 million	15 (11.6)	27.87±2.82		
Current drinking	Yes	78 (60.5)	26.95±3.79	1.12	.263
	No	51 (39.5)	26.15±4.10		
Current smoking	Yes	14 (10.9)	25.78±3.81	–0.86	.392
	No	115 (89.1)	26.74±3.94		
Menopause	Yes	11 (8.5)	26.74±3.88	1.05	.298
	No	118 (91.5)	25.45±4.27		
Age at first sexual intercourse (year)		20.80±3.09			
	<20^a^	39 (30.2)	27.67±3.47	7.38	.001
	20–24^b^	76 (58.9)	26.74±3.80		(a, b>c)^†^
	≥25^c^	14 (10.9)	23.21±4.10		
No. of sexual partners during lifetime		4.74±4.20			
	1	24 (18.6)	26.04±3.95	1.05	.354
	2–4	53 (41.1)	26.32±4.07		
	≥5	52 (40.3)	27.23±3.73		
Current condom use	Yes	34 (26.4)	28.09±2.88	3.03	.003
	No	95 (73.6)	26.11±4.12		
Frequency of sexual intercourse	Less than once a month	62 (48.1)	26.68±3.74	0.59	.619
	Once a week	55 (42.6)	26.33±4.14		
	2–3 times a week	11 (8.5)	27.64±3.98		
	4 or more times a week	1 (0.8)	30.00		
Pap test	Every 6 months	35 (27.1)	26.77±4.06	1.22	.306
	Every 1 year	45 (34.9)	27.38±3.53		
	Every 2 years	40 (31.0)	25.80±4.14		
	More than 3 years	9 (7.0)	26.11±4.07		
HPV vaccination	Yes	66 (51.2)	27.68±3.04	3.19	.002
	No	63 (48.8)	25.54±4.43		
Induced abortion	Yes	39 (30.2)	26.79±3.73	–0.30	.763
	No	90 (69.8)	26.57±4.02		
History of childbirth	Yes	72 (55.8)	26.24±4.09	1.30	.194
	No	57 (44.2)	27.14±3.66		
HPV type	Low risk^a^	21 (16.3)	24.33±4.95	4.79	.010
	High risk^b^	66 (51.2)	26.89±3.77		(b, c>a)^†^
	Both^c^	42 (32.6)	27.38±3.16		

HPV: human papillomavirus; KRW: Korean won (1 million KRW=roughly 800 US dollars).

† Scheffé test.

**Table 2.** Summary of cervical cancer knowledge, HPV knowledge, uncertainty, self-efficacy, intention to engage in cervical cancer preventive behavior (N=129)

Variable	Possible range	Minimum	Maximum	Mean±SD
Intention to engage in cervical cancer preventive behavior	1–5	3	5	4.43±0.65
Cervical cancer knowledge	0–8	0	8	4.87±1.86
HPV knowledge	0–20	0	18	10.04±4.36
Uncertainty	1–5	1.26	3.91	2.42±0.52
Self-efficacy	1–5	1.79	5	3.90±0.70

HPV: human papillomavirus.

**Table 3.** Relationships among cervical cancer knowledge, HPV knowledge, uncertainty, self-efficacy and intention to engage in cervical cancer preventive behavior (N=129)

Variable	r(*p*)			
	Intention to engage in cervical cancer preventive behavior	Cervical cancer knowledge	HPV knowledge	Uncertainty
Intention to engage in cervical cancer preventive behavior	1			
Cervical cancer knowledge	.13 (.138)	1		
HPV knowledge	.22 (.012)	.63 (<.001)	1	
Uncertainty	–.09 (.321)	–.21 (.016)	–.21 (.015)	1
Self-efficacy	.42 (<.001)	.07 (.401)	.14 (.108)	–.33 (<.001)

HPV: human papillomavirus.

**Table 4.** Linear regression on factors associated with the intention to engage in cervical cancer preventive behavior (N=129)

Variable	B	SE	β	t	*p*
(Constant)	10.47	2.83		3.71	<.001
Cervical cancer knowledge	0.07	0.20	.04	0.33	.741
HPV knowledge	–0.02	0.09	–.02	–0.22	.830
Uncertainty	0.01	0.03	.04	0.52	.601
Self-efficacy	0.11	0.02	.46	5.80	<.001
Age at first sexual intercourse (year)^‡^					
<20	3.82	1.02	.45	3.74	<.001
20–24	2.32	0.97	.29	2.41	.018
HPV type‡					
High-risk	2.00	0.88	.26	2.26	.026
Both high- and low-risk	2.14	0.90	.26	2.37	.019
HPV vaccination^‡^	1.63	0.60	.21	2.72	.007
Current condom use^‡^	0.57	0.71	.06	0.80	.427
Adjusted R^2^=.35, F=7.76, *p*<.001					

HPV: human papillomavirus.

‡ The reference groups were age at first sexual intercourse (≥25), HPV type (low-risk), HPV vaccination (no), and current condom use (no).
